# The genome sequence of the small wasp-sawfly, *Tenthredo distinguenda *(R. Stein, 1885)

**DOI:** 10.12688/wellcomeopenres.19523.1

**Published:** 2023-10-12

**Authors:** Steven Falk, Andrew Green, University of Oxford and Wytham Woods Genome Acquisition Lab, Darwin Tree of Life Barcoding collective, Wellcome Sanger Institute Tree of Life programme, Wellcome Sanger Institute Scientific Operations: DNA Pipelines collective, Tree of Life Core Informatics collective, Darwin Tree of Life Consortium

**Affiliations:** 1 Independent researcher, Kenilworth, England, UK; 2 Sawfly Recording Scheme, Bedford, England, UK

**Keywords:** Tenthredo distinguenda, small wasp-sawfly, genome sequence, chromosomal, Hymenoptera

## Abstract

We present a genome assembly from an individual male *Tenthredo distinguenda* (the small wasp-sawfly; Arthropoda; Insecta; Hymenoptera; Tenthredinidae). The genome sequence is 229.4 megabases in span. Most of the assembly is scaffolded into 9 chromosomal pseudomolecules. The mitochondrial genome has also been assembled and is 31.6 kilobases in length. Gene annotation of this assembly on Ensembl identified 11,332 protein coding genes.

## Species taxonomy

Eukaryota; Metazoa; Eumetazoa; Bilateria; Protostomia; Ecdysozoa; Panarthropoda; Arthropoda; Mandibulata; Pancrustacea; Hexapoda; Insecta; Dicondylia; Pterygota; Neoptera; Endopterygota; Hymenoptera; Tenthredinoidea; Tenthredinidae; Tenthredininae; *Tenthredo*; *Tenthredo distinguenda* (R. Stein, 1885) (NCBI:txid1385102).

## Background

The *Tenthredo* genus is large, with over one thousand species distributed across the Holarctic region and Oriental regions. There are 30 species present in Britain. Within this genus, numerous species groups and subspecies have been identified. *Tenthredo distinguenda* (R. Stein, 1885) falls within the subgenus *Zonuledo*, together in Britain with *Tenthredo amoena* (Gravenhorst, 1807).

The subspecies *Tenthredo distinguenda distinguenda* (R. Stein, 1885) is distributed across Europe and is a comparatively small (8.5 to 9.5 mm.), black *Tenthredo*, marked with yellow on the head, antennae, pronotum, tegulae, abdomen and legs. The subspecies *Tenthredo distinguenda hyrcana ( Benson, 1968)* occurs in Eastern Europe, Turkey and Iran. However, the status of *hyrcana* is still in discussion. In Britain, adults can be distinguished from the similar *T. amoena* by a combination of the densely punctured and dull mesepisternum, entirely yellow tegulae and antennal scapes marked with black on the outer face. Little is known about the ecology of the species, but many *Tenthredo* are predatory species that contribute to pest control. The larvae feed on *Hypericum perforatum*, Perforate St John’s Wort, and as such are not considered to be a pest of agricultural or horticultural significance ( Macek *et al.*, 2020). The species is univoltine, with adults on the wing from May to July.

Although the species boundaries remain unclear, in Europe and North Africa there are currently eight named species in the subgenus. These species are morphologically very similar with high levels of intra-species character variability. Indeed, the genital structures of both males and females exhibit variability and are not considered reliable identification characteristics ( Taeger, 1991). Phylogenetic classification of the *Zonuledo* subgenus based on morphology is problematic due to the lack of obvious synapomorphic features. In BOLD, COI barcoding produces four well-defined clusters, namely *Tenthredo flavipennis * (Brull, 1832), *Tenthredo zonula * (Klug, 1817), *T. distinguenda* and *T. amoena*. One specimen of *T. distinguenda hyrcana* from Armenia falls into a separate BIN ABU8418. A further specimen from Iran, which appears from images to be *T. distinguenda*, is close to *T. zonula* (in this case, contamination cannot be excluded). The remaining *T. distinguenda* specimens all fall within BIN ABU8417 (pers. comm. Taeger, 2023). In the southern parts of its range, *T. distinguenda* appears variable in colour and the current species *Tenthredo lacourti* ( Taeger, 1991), *Tenthredo kervillei* (Konow, 1907) and *Tenthredo berberensis* (Lacourt, 1986) could be forms or subspecies of *T. distinguenda* (pers. comm. Taeger, 2023).

There are no previously barcoded specimens from Britain. Knowledge of sawfly evolution will benefit from the comparative analysis of genomes from closely and distantly related species. This male specimen from Wytham Woods, England matches the description of *T. distinguenda distinguenda* using the characteristics in Benson’s key ( Benson, 1952) and the publication of the complete gene sequence will help our understanding of the phylogeny of this group.

## Genome sequence report

**Figure 1.**
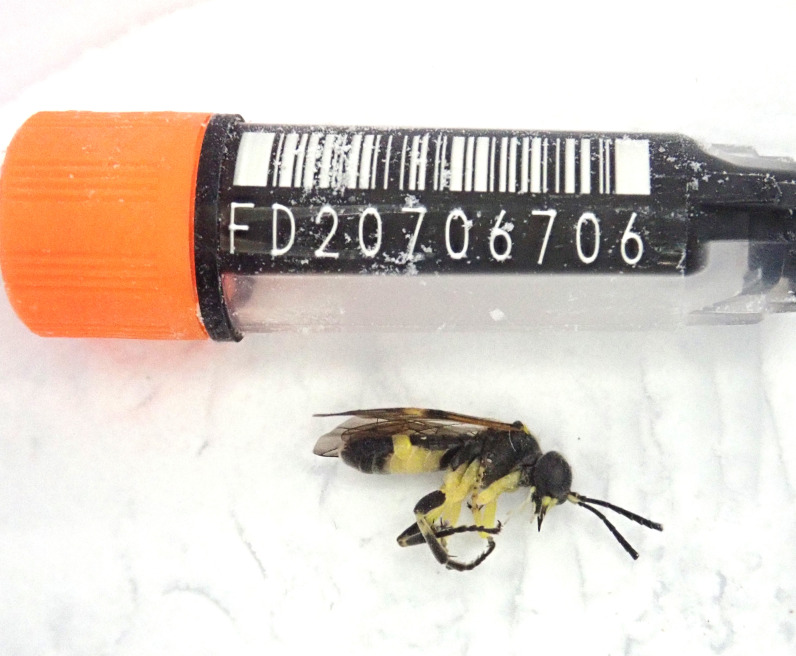
Photograph of the *Tenthredo distinguenda* (iyTenDist1) specimen used for genome sequencing.

The genome was sequenced from one male *Tenthredo distinguenda* ( Figure 1) collected from Wytham Woods, Oxfordshire, UK (51.76, –1.33). A total of 81-fold coverage in Pacific Biosciences single-molecule HiFi long reads was generated. Primary assembly contigs were scaffolded with chromosome conformation Hi-C data. Manual assembly curation corrected 14 missing joins or mis-joins, reducing the scaffold number by 8.65%, and increasing the scaffold N50 by 54.96%.

**Figure 2.**
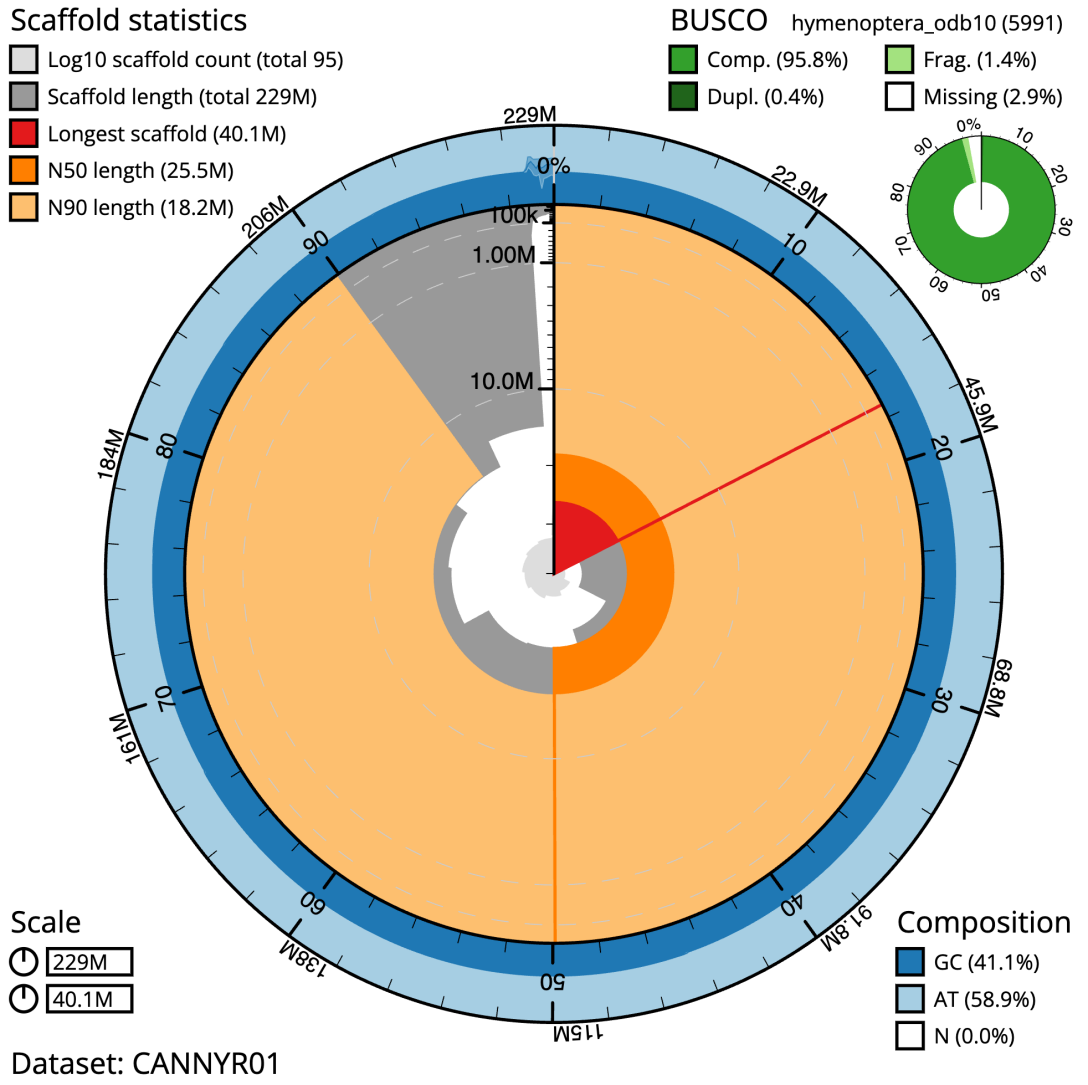
Genome assembly of *Tenthredo distinguenda*, iyTenDist1.1: metrics. The BlobToolKit Snailplot shows N50 metrics and BUSCO gene completeness. The main plot is divided into 1,000 size-ordered bins around the circumference with each bin representing 0.1% of the 229,413,372 bp assembly. The distribution of scaffold lengths is shown in dark grey with the plot radius scaled to the longest scaffold present in the assembly (40,074,581 bp, shown in red). Orange and pale-orange arcs show the N50 and N90 scaffold lengths (25,528,205 and 18,198,451 bp), respectively. The pale grey spiral shows the cumulative scaffold count on a log scale with white scale lines showing successive orders of magnitude. The blue and pale-blue area around the outside of the plot shows the distribution of GC, AT and N percentages in the same bins as the inner plot. A summary of complete, fragmented, duplicated and missing BUSCO genes in the hymenoptera_odb10 set is shown in the top right. An interactive version of this figure is available at https://blobtoolkit.genomehubs.org/view/iyTenDist1.1/dataset/CANNYR01/snail.

The final assembly has a total length of 229.4 Mb in 95 sequence scaffolds with a scaffold N50 of 25.5 Mb ( Table 1). Most (98.81%) of the assembly sequence was assigned to 9 chromosomal-level scaffolds. Chromosome-scale scaffolds confirmed by the Hi-C data are named in order of size ( Figure 2– Figure 5; Table 2). The mitochondrial genome was also assembled and can be found as a contig within the multifasta file of the genome submission.

**Table 1.** Genome data for *Tenthredo distinguenda*, iyTenDist1.1.

Project accession data		
Assembly identifier	iyTenDist1.1	
Species	*Tenthredo distinguenda*	
Specimen	iyTenDist1	
NCBI taxonomy ID	1385102	
BioProject	PRJEB55569	
BioSample ID	SAMEA10167068	
Isolate information	iyTenDist1, male, whole organism (DNA sequencing) iyTenDist2, whole organism (Hi-C scaffolding)	
Assembly metrics *		*Benchmark*
Consensus quality (QV)	70.7	*≥ 50*
*k*-mer completeness	100%	*≥ 95%*
BUSCO **	C:95.8%[S:95.3%,D:0.4%], F:1.4%,M:2.9%,n:5,991	*C ≥ 95%*
Percentage of assembly mapped to chromosomes	98.81%	*≥ 95%*
Sex chromosomes	-	*localised homologous pairs*
Organelles	Mitochondrial genome assembled	*complete single alleles*
Raw data accessions		
PacificBiosciences SEQUEL II	ERR10115638	
Hi-C Illumina	ERR10123711	
Genome assembly		
Assembly accession	GCA_947538915.1	
Span (Mb)	229.4	
Number of contigs	111	
Contig N50 length (Mb)	14.1	
Number of scaffolds	94	
Scaffold N50 length (Mb)	25.5	
Longest scaffold (Mb)	40.1	
Genome annotation		
Number of protein-coding genes	11,332	
Number of non-coding genes	1,691	
Number of gene transcripts	19,107	

* Assembly metric benchmarks are adapted from column VGP-2020 of “Table 1: Proposed standards and metrics for defining genome assembly quality” from ( Rhie *et al.*, 2021). ** BUSCO scores based on the hymenoptera_odb10 BUSCO set using v5.3.2. C = complete [S = single copy, D = duplicated], F = fragmented, M = missing, n = number of orthologues in comparison. A full set of BUSCO scores is available at https://blobtoolkit.genomehubs.org/view/iyTenDist1.1/dataset/CANNYR01/busco.

**Figure 3.**
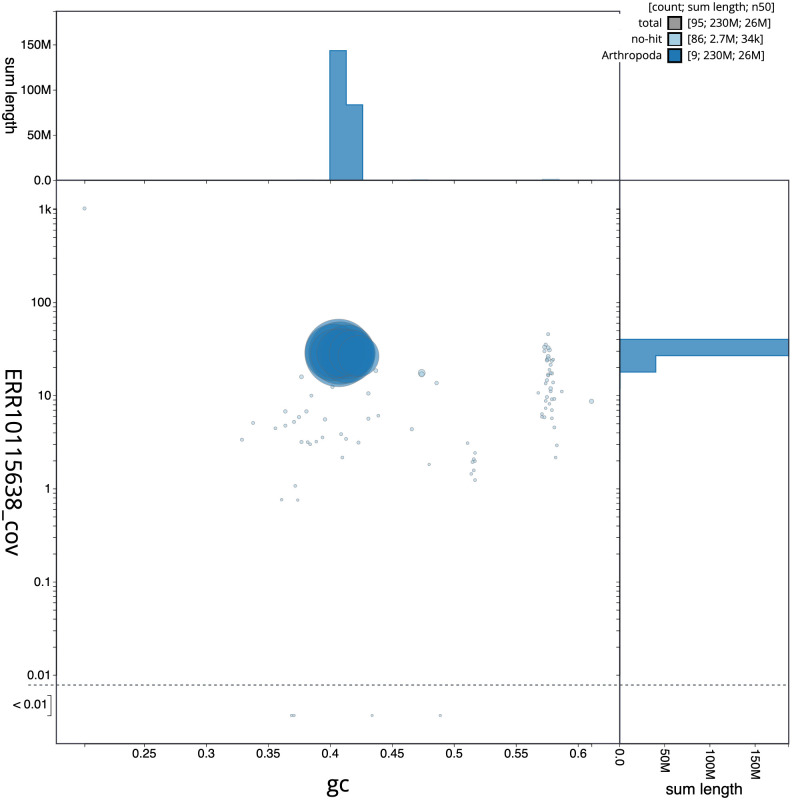
Genome assembly of *Tenthredo distinguenda*, iyTenDist1.1: BlobToolKit GC-coverage plot. Scaffolds are coloured by phylum. Circles are sized in proportion to scaffold length. Histograms show the distribution of scaffold length sum along each axis. An interactive version of this figure is available at https://blobtoolkit.genomehubs.org/view/iyTenDist1.1/dataset/CANNYR01/blob.

**Figure 4.**
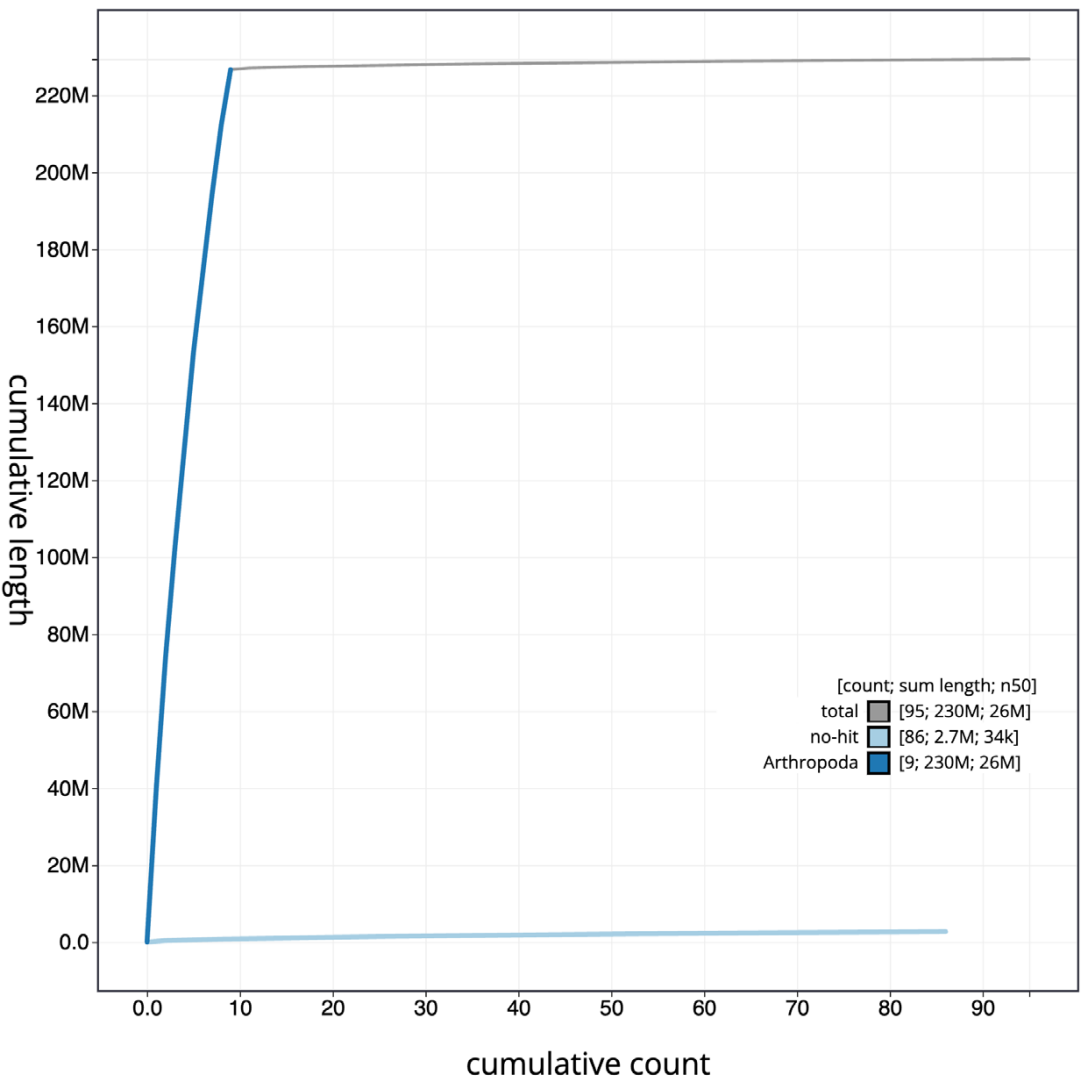
Genome assembly of *Tenthredo distinguenda*, iyTenDist1.1: BlobToolKit cumulative sequence plot. The grey line shows cumulative length for all scaffolds. Coloured lines show cumulative lengths of scaffolds assigned to each phylum using the buscogenes taxrule. An interactive version of this figure is available at https://blobtoolkit.genomehubs.org/view/iyTenDist1.1/dataset/CANNYR01/cumulative.

**Figure 5.**
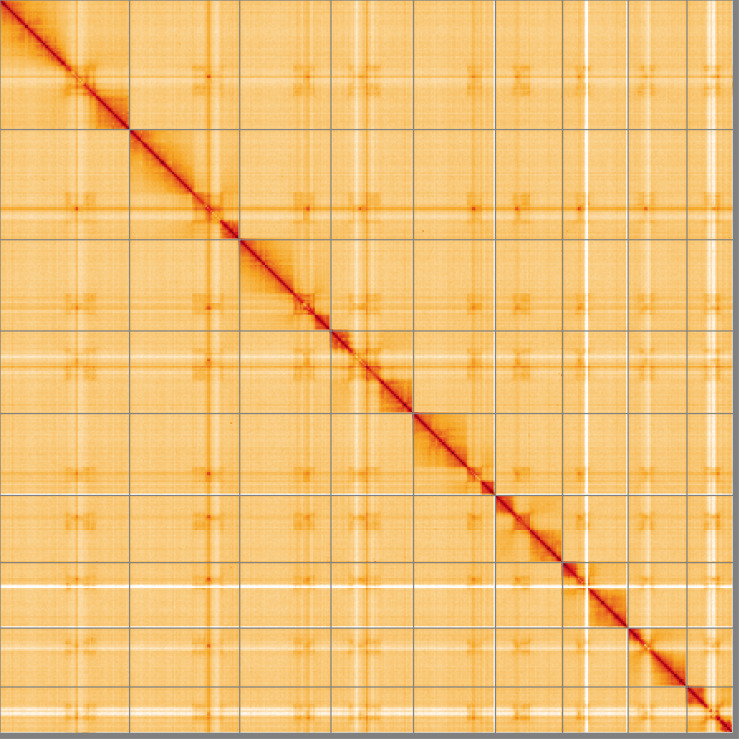
Genome assembly of *Tenthredo distinguenda*, iyTenDist1.1: Hi-C contact map of the iyTenDist1.1 assembly, visualised using HiGlass. Chromosomes are shown in order of size from left to right and top to bottom. An interactive version of this figure may be viewed at https://genome-note-higlass.tol.sanger.ac.uk/l/?d=GTiO0K6pQQubqIdEMilHsQ.

**Table 2.** Chromosomal pseudomolecules in the genome assembly of *Tenthredo distinguenda*, iyTenDist1.

INSDC accession	Name	Length (Mb)	GC%
OX384530.1	1	40.07	40.5
OX384531.1	2	34.06	41
OX384532.1	3	28.2	40.5
OX384533.1	4	25.53	41.5
OX384534.1	5	25.33	41.5
OX384535.1	6	20.76	40.5
OX384536.1	7	20.25	41
OX384537.1	8	18.2	42
OX384538.1	9	14.31	42.5
OX384539.1	MT	0.03	20

The estimated Quality Value (QV) of the final assembly is 70.7 with *k*-mer completeness of 100%, and the assembly has a BUSCO v5.3.2 completeness of 95.8% (single = 95.3%, duplicated = 0.4%), using the hymenoptera_odb10 reference set ( *n* = 5,991).

Metadata for specimens, spectral estimates, sequencing runs, contaminants and pre-curation assembly statistics can be found at https://links.tol.sanger.ac.uk/species/1385102.

## Genome annotation report

The *Tenthredo distinguenda* genome assembly (GCA_947538915.1) was annotated using the Ensembl rapid annotation pipeline ( Table 1; https://rapid.ensembl.org/Tenthredo_distinguenda_GCA_947538915.1/Info/Index). The resulting annotation includes 19,107 transcribed mRNAs from 11,332 protein-coding and 1,691 non-coding genes.

## Methods

### Sample acquisition and nucleic acid extraction

Two *Tenthredo distinguenda* specimens were collected from Wytham Woods, Oxfordshire (biological vice-county Berkshire), UK (latitude 51.76, longitude –1.33) on 2021-05-31. The specimens were netted in a woodland habitat by Steven Falk (independent researcher), and identified by the same person. The specimens were preserved on dry ice. The specimen used for genome sequencing was specimen number Ox001514, iyTenDist1 ( Figure 1), while the second specimen, Ox001520, iyTenDist2 was used for Hi-C scaffolding.

DNA was extracted at the Tree of Life laboratory, Wellcome Sanger Institute (WSI). The iyTenDist1 sample was weighed and dissected on dry ice. Whole organism tissue was disrupted using a Nippi Powermasher fitted with a BioMasher pestle. High molecular weight (HMW) DNA was extracted using the Qiagen MagAttract HMW DNA extraction kit. HMW DNA was sheared into an average fragment size of 12–20 kb in a Megaruptor 3 system with speed setting 30. Sheared DNA was purified by solid-phase reversible immobilisation using AMPure PB beads with a 1.8X ratio of beads to sample to remove the shorter fragments and concentrate the DNA sample. The concentration of the sheared and purified DNA was assessed using a Nanodrop spectrophotometer and Qubit Fluorometer and Qubit dsDNA High Sensitivity Assay kit. Fragment size distribution was evaluated by running the sample on the FemtoPulse system.

### Sequencing

Pacific Biosciences HiFi circular consensus DNA sequencing libraries were constructed according to the manufacturers’ instructions. DNA sequencing was performed by the Scientific Operations core at the WSI on the Pacific Biosciences SEQUEL II (HiFi) instrument. Hi-C data were also generated from whole tissue of iyTenDist2 using the Arima2 kit and sequenced on the Illumina NovaSeq 6000 instrument.

### Genome assembly, curation and evaluation

Assembly was carried out with Hifiasm ( Cheng *et al.*, 2021) and haplotypic duplication was identified and removed with purge_dups ( Guan *et al.*, 2020). The assembly was then scaffolded with Hi-C data ( Rao *et al.*, 2014) using YaHS ( Zhou *et al.,* 2023). The assembly was checked for contamination and corrected as described previously ( Howe *et al.*, 2021). Manual curation was performed using HiGlass ( Kerpedjiev *et al.*, 2018) and Pretext ( Harry, 2022). The mitochondrial genome was assembled using MitoHiFi ( Uliano-Silva *et al.*, 2022), which runs MitoFinder ( Allio *et al.*, 2020) or MITOS ( Bernt *et al.*, 2013) and uses these annotations to select the final mitochondrial contig and to ensure the general quality of the sequence.

A Hi-C map for the final assembly was produced using bwa-mem2 ( Vasimuddin *et al.*, 2019) in the Cooler file format ( Abdennur & Mirny, 2020). To assess the assembly metrics, the *k*-mer completeness and QV consensus quality values were calculated in Merqury ( Rhie *et al.*, 2020). This work was done using Nextflow ( Di Tommaso *et al.*, 2017) DSL2 pipelines “sanger-tol/readmapping” ( Surana *et al.,* 2023a) and “sanger-tol/genomenote” ( Surana *et al.,* 2023b). The genome was analysed within the BlobToolKit environment ( Challis *et al.*, 2020) and BUSCO scores ( Manni *et al.*, 2021; Simão *et al.*, 2015) were calculated.

Table 3 contains a list of relevant software tool versions and sources.

**Table 3.** Software tools: versions and sources.

Software tool	Version	Source
BlobToolKit	4.0.7	https://github.com/blobtoolkit/blobtoolkit
BUSCO	5.3.2	https://gitlab.com/ezlab/busco
Hifiasm	0.16.1-r375	https://github.com/chhylp123/hifiasm
HiGlass	1.11.6	https://github.com/higlass/higlass
Merqury	MerquryFK	https://github.com/thegenemyers/MERQURY.FK
MitoHiFi	2.3	https://github.com/marcelauliano/MitoHiFi
PretextView	0.2	https://github.com/wtsi-hpag/PretextView
sanger-tol/genomenote	v1.0	https://github.com/sanger-tol/genomenote
sanger-tol/readmapping	1.1.0	https://github.com/sanger-tol/readmapping/tree/1.1.0
YaHS	yahs-1.1.91eebc2	https://github.com/c-zhou/yahs

### Genome annotation

The Ensembl gene annotation system ( Aken *et al.*, 2016) was used to generate annotation for the *Tenthredo distinguenda* assembly (GCA_947538915.1). Annotation was created primarily through alignment of transcriptomic data to the genome, with gap filling via protein-to-genome alignments of a select set of proteins from UniProt ( UniProt Consortium, 2019).

### Wellcome Sanger Institute – Legal and Governance

The materials that have contributed to this genome note have been supplied by a Darwin Tree of Life Partner. The submission of materials by a Darwin Tree of Life Partner is subject to the **‘Darwin Tree of Life Project Sampling Code of Practice’**, which can be found in full on the Darwin Tree of Life website here. By agreeing with and signing up to the Sampling Code of Practice, the Darwin Tree of Life Partner agrees they will meet the legal and ethical requirements and standards set out within this document in respect of all samples acquired for, and supplied to, the Darwin Tree of Life Project.

Further, the Wellcome Sanger Institute employs a process whereby due diligence is carried out proportionate to the nature of the materials themselves, and the circumstances under which they have been/are to be collected and provided for use. The purpose of this is to address and mitigate any potential legal and/or ethical implications of receipt and use of the materials as part of the research project, and to ensure that in doing so we align with best practice wherever possible. The overarching areas of consideration are:

- Ethical review of provenance and sourcing of the material
- Legality of collection, transfer and use (national and international)

Each transfer of samples is further undertaken according to a Research Collaboration Agreement or Material Transfer Agreement entered into by the Darwin Tree of Life Partner, Genome Research Limited (operating as the Wellcome Sanger Institute), and in some circumstances other Darwin Tree of Life collaborators.

## Data Availability

European Nucleotide Archive: *Tenthredo distinguenda*. Accession number PRJEB55569; https://identifiers.org/ena.embl/PRJEB55569. ( Wellcome Sanger Institute, 2022) The genome sequence is released openly for reuse. The *Tenthredo distinguenda* genome sequencing initiative is part of the Darwin Tree of Life (DToL) project. All raw sequence data and the assembly have been deposited in INSDC databases. Raw data and assembly accession identifiers are reported in Table 1.

## Author information

Members of the University of Oxford and Wytham Woods Genome Acquisition Lab are listed here: https://doi.org/10.5281/zenodo.4789928.

Members of the Darwin Tree of Life Barcoding collective are listed here: https://doi.org/10.5281/zenodo.4893703.

Members of the Wellcome Sanger Institute Tree of Life programme are listed here: https://doi.org/10.5281/zenodo.4783585.

Members of Wellcome Sanger Institute Scientific Operations: DNA Pipelines collective are listed here: https://doi.org/10.5281/zenodo.4790455.

Members of the Tree of Life Core Informatics collective are listed here: https://doi.org/10.5281/zenodo.5013541.

Members of the Darwin Tree of Life Consortium are listed here: https://doi.org/10.5281/zenodo.4783558.
