# Supplementary material for: The genome sequence of the small wasp-sawfly, Tenthredo distinguenda (R. Stein, 1885)
Source: Wellcome Open Res. 2023 Oct 12;8:459. [Version 1] doi: 10.12688/wellcomeopenres.19523.1 (PMC11109533; doi:10.12688/wellcomeopenres.19523.1)
Supplement: Peer review history

# Reviewer report: Reviewer response for version 1

**DOI:** 10.21956/wellcomeopenres.21626.r72611

**Published:** 2024-05-13

**Authors:** Marko Mutanen

**Affiliations:** 1 University of Oulu, Oulu, Finland

This article provides a genome sequence for a sawfly species *Tenthredo distinguenda*. It belongs to a diverse family *Tenthredinidae*, its subfamily *Tenthredininae* and the species-rich genus *Tenthredo*. Well-assembled and high-quality genomes for sawflies are badly needed, and this work is a welcomed addition. Sawflies have turned out to be taxonomically an unusually difficult group with unusual levels of DNA barcode-sharing, frequent presence of multiple functional looking full-length barcodes within individuals and often elusive differences between species. It is likely that genomic approaches are required to understand their taxonomy. *Tenthredo* is an excellent example as even within Europe there are many examples of cases where the species boundaries remain unclear.

 The Introduction has some focus on comparing the target species to *T. amoena*, probably because it is the closest relative of *T. distinguenda* in UK. However, I believe the closest relative is *T. zonula*. Fortunately, this species is also considered and the barcode differences of the subgenus are compared.

 The produced genome shows metrics that to my (slightly limited) understanding suggest that the new genome is of high-quality. For example, BUSCO gene completeness is of high level. I cannot assess all technical details of this work, yet I am confident that that the genome is overall of very high-quality, for which reason I warmly recommend this genome work.

Are sufficient details of methods and materials provided to allow replication by others?

Yes

Is the rationale for creating the dataset(s) clearly described?

Yes

Are the datasets clearly presented in a useable and accessible format?

Yes

Are the protocols appropriate and is the work technically sound?

Yes

Reviewer Expertise:

I am an insect taxonomist with species expertise especially on Lepidoptera and sawflies. I am predominantly using genetic and genomic methods in my research. While I have experience of various genomic tools, my experience in building full genomes is limited.

I confirm that I have read this submission and believe that I have an appropriate level of expertise to confirm that it is of an acceptable scientific standard.

# Reviewer report: Reviewer response for version 1

**DOI:** 10.21956/wellcomeopenres.21626.r72612

**Published:** 2024-05-11

**Authors:** Denilce Lopes

**Affiliations:** 1 Departamento de Biologia Geral, Universidade Federal de Vicosa, Viçosa, State of Minas Gerais, Brazil

The authors present genomic sequencing, nuclear and mytocondrial data from the wasp-sawfly, Tenthredo distinguenda. The analysis methods are adequate, similar to those presented by other works within the Darwin Tree of Life Barcoding collective project. The indicators show high data quality.

Are sufficient details of methods and materials provided to allow replication by others?

Yes

Is the rationale for creating the dataset(s) clearly described?

Yes

Are the datasets clearly presented in a useable and accessible format?

Yes

Are the protocols appropriate and is the work technically sound?

Yes

Reviewer Expertise:

Cytogenetic and genomic

I confirm that I have read this submission and believe that I have an appropriate level of expertise to confirm that it is of an acceptable scientific standard.

# Reviewer report: Reviewer response for version 1

**DOI:** 10.21956/wellcomeopenres.21626.r76757

**Published:** 2024-04-05

**Authors:** Saskia Wutke

**Affiliations:** 1 Department of Environmental and Biological Sciences, University or Eastern Finland, Joensuu, Finland

This data note describes the genome of the sawfly *Tenthredo distinguenda*. The genome was assembled using PacBio HiFi data and scaffolded with Hi-C data. Annotation was performed based on transcriptome data. The methods are state-of-the-art and follow the usual DToL procedure. The final chromosome-level genome assembly is 229.4 Mb long. With a scaffold N50 of 25.5 Mb and a total number of 95 scaffolds, the assembly is of good quality and provides a valuable addition to the growing list of sawfly genomes. 

 Nevertheless, I have a few comments regarding the methods and background. 

**BACKGROUND:**

 - It is mentioned that *T. distinguenda* is a predatory species that contributes to pest control, but not explained in more detail which pests would be controlled. 

 - The structure of the background section is rather confusing, jumping from subspecies details to related species. Although I appreciate the taxonomic details, the background would benefit from re-ordering the paragraphs. 

**METHODS:**

  - For better reproducibility, the specific settings of the software would be good to know, in addition to the versions (table 3). 

  - I assume that the males of this species are haploid, like it is the case for most Hymenoptera. I am wondering how much the purge_dups step after initial assembly improves the assembly. This could be easily checked by calculating (and providing) the usual assembly stats and completeness measures (QV, BUSCO). 

 - Please provide more details on the transcriptome data used for annotation (source of the data and accession numbers) and the protein set from UniProt.

Are sufficient details of methods and materials provided to allow replication by others?

Partly

Is the rationale for creating the dataset(s) clearly described?

Yes

Are the datasets clearly presented in a useable and accessible format?

Yes

Are the protocols appropriate and is the work technically sound?

Yes

Reviewer Expertise:

Evolutionary genomics, phylogenomics, Hymenoptera evolution

I confirm that I have read this submission and believe that I have an appropriate level of expertise to confirm that it is of an acceptable scientific standard, however I have significant reservations, as outlined above.
